# Transmembrane Peptides as a New Strategy to Inhibit Neuraminidase-1 Activation

**DOI:** 10.3389/fcell.2020.611121

**Published:** 2020-12-16

**Authors:** Camille Albrecht, Andrey S. Kuznetsov, Aline Appert-Collin, Zineb Dhaideh, Maïté Callewaert, Yaroslav V. Bershatsky, Anatoly S. Urban, Eduard V. Bocharov, Dominique Bagnard, Stéphanie Baud, Sébastien Blaise, Béatrice Romier-Crouzet, Roman G. Efremov, Manuel Dauchez, Laurent Duca, Marc Gueroult, Pascal Maurice, Amar Bennasroune

**Affiliations:** ^1^Université de Reims Champagne-Ardenne, Reims, France; ^2^CNRS UMR 7369, Matrice Extracellulaire et Dynamique Cellulaire (MEDyC), Reims, France; ^3^Shemyakin-Ovchinnikov Institute of Bioorganic Chemistry, Russian Academy of Sciences, Moscow, Russia; ^4^Higher School of Economics, Moscow, Russia; ^5^Moscow Institute of Physics and Technology, National Research University, Dolgoprudny, Russia; ^6^CNRS UMR 7312, Institut de Chimie Moléculaire de Reims, Reims, France; ^7^Université de Strasbourg, Strasbourg, France; ^8^INSERM U1119 Biopathologie de la Myéline, Neuroprotection et Stratégies Thérapeutiques, Labex Medalis, Fédération de Médecine Translationnelle de Strasbourg, Strasbourg, France; ^9^Plateau de Modélisation Moléculaire Multi-échelle, Reims, France

**Keywords:** neuraminidase-1, sialidase activity, transmembrane domain, membrane protein dimerization, interfering peptides

## Abstract

Sialidases, or neuraminidases, are involved in several human disorders such as neurodegenerative, infectious and cardiovascular diseases, and cancers. Accumulative data have shown that inhibition of neuraminidases, such as NEU1 sialidase, may be a promising pharmacological target, and selective inhibitors of NEU1 are therefore needed to better understand the biological functions of this sialidase. In the present study, we designed interfering peptides (IntPep) that target a transmembrane dimerization interface previously identified in human NEU1 that controls its membrane dimerization and sialidase activity. Two complementary strategies were used to deliver the IntPep into cells, either flanked to a TAT sequence or non-tagged for solubilization in detergent micelles. Combined with molecular dynamics simulations and heteronuclear nuclear magnetic resonance (NMR) studies in membrane-mimicking environments, our results show that these IntPep are able to interact with the dimerization interface of human NEU1, to disrupt membrane NEU1 dimerization and to strongly decrease its sialidase activity at the plasma membrane. In conclusion, we report here new selective inhibitors of human NEU1 of strong interest to elucidate the biological functions of this sialidase.

## Introduction

Sialidases, or neuraminidases, are glycosidases responsible for the cleavage of terminal sialic acid residues from glycoproteins, glycolipids, and oligosaccharides (Monti et al., 2010). These enzymes are widely distributed among species and found in viruses, protozoa, bacteria, fungi, and vertebrates (Giacopuzzi et al., 2012). Four types of mammalian sialidases, encoded by different genes, have been described with distinct substrate specificity and subcellular localization: NEU1, NEU2, NEU3, and NEU4 (Miyagi and Yamaguchi, 2012). Sialidases have been involved in a wide range of human disorders, including neurodegenerative disorders, cancers, infectious, and cardiovascular diseases (Glanz et al., 2019).

Emerging data have demonstrated that NEU1, firstly identified in lysosomes (Bonten et al., 1996), is also found at the plasma membrane and regulates a myriad of membrane receptors by desialylation resulting in either activation or inhibition of the receptors (Hinek et al., 2008; Uemura et al., 2009; Amith et al., 2010; Jayanth et al., 2010; Lillehoj et al., 2012; Blaise et al., 2013; Dridi et al., 2013; Lee et al., 2014; Kawecki et al., 2019). At the plasma membrane, NEU1 also associates with the elastin-binding protein (EBP) and the carboxypeptidase protective protein/cathepsin A (PPCA) forming the elastin receptor complex (ERC). NEU1 is required for elastogenesis and signal transduction through this receptor (Hinek et al., 2006; Duca et al., 2007; Rusciani et al., 2010) and for the biological effects mediated by the elastin-derived peptides on atherosclerosis (Gayral et al., 2014; Kawecki et al., 2019), thrombosis (Kawecki et al., 2014), insulin resistance (Blaise et al., 2013) and non-alcoholic steatohepatitis (Romier et al., 2018). Finally, recent findings have also highlighted a critical role of NEU1 in immune thrombocytopenia induced by anti-GPIbα autoantibodies by a mechanism involving Fc-independent platelet activation, NEU1 translocation to the plasma membrane, desialylation, and platelet clearance in the liver via hepatocyte Ashwell–Morell receptors (Li et al., 2015). Together, these recent findings make NEU1 a pharmacological target of high potential and inhibitors of human NEU1 are therefore urgently needed.

There are still no commercially available inhibitors that are selective for NEU1, especially due to the lack of structural information for NEU1. Human NEUs are assumed to share a common β-propeller structure organized in six blades with highly conserved motifs implicated in their catalytic activity (Magesh et al., 2006). The role of NEU1 was therefore investigated mainly using the broad-spectrum sialidase inhibitor DANA, or inhibitors of bacterial or viral NEUs, such as zanamivir or oseltamivir. However, bacterial or viral NEU inhibitors have broad or weak activity against human NEUs (Hata et al., 2008; Richards et al., 2018). To date, two selective inhibitors of human NEU1 have been reported in the literature, based on modifications of the DANA scaffold: the C9-amido analog of DANA (C9-BA-DANA) which shows micromolar IC_50_ against human NEU1 (Magesh et al., 2008) and the C5-hexanamido-C9-acetamido analog with a *K*_*i*_ of 53 ± 5 nM and 330-fold selectivity (Guo et al., 2018). Only C9-BA-DANA has been tested *in vitro* and *in vivo*. Data have shown that C9-BA-DANA efficiently inhibits endogenous and ectopically expressed sialidase activity and established NEU1-mediated bioactivities in human airway epithelia, lung microvascular endothelia, fibroblasts *in vitro* and murine lungs *in vivo* (Hyun et al., 2016).

By combining molecular biology and biochemical analyses with structural biophysics and computational approaches, we recently identified two segments in human NEU1 as potential transmembrane (TM) domains (Maurice et al., 2016). In membrane-mimicking environments, the corresponding peptides were shown to form stable α-helices and the peptide covering the segment 316–333 (TM2) of NEU1 was suited for self-association. *In vitro* experiments further confirmed the ability of membrane NEU1 (mNEU1) to dimerize and importantly, introduction of point mutations within TM2 was associated with substantial disruption of mNEU1 dimerization and decrease of its sialidase activity (Maurice et al., 2016). These results therefore strongly suggested that dimerization of mNEU1 controls its catalytic activity and that targeting this dimerization interface may represent an original strategy for selective inhibition of human mNEU1.

In the present study, we designed interfering peptides (IntPep) that target the dimerization interface located in the TM2 domain of mNEU1 and evaluated their effects on its sialidase activity. Two complementary strategies were used to deliver the IntPep into cells; either flanked to the GRKKRRQRRRPQ TAT sequence (Bechara and Sagan, 2013) or non-tagged for solubilization in lithium dodecyl sulfate (LDS) micelles as previously described (Rangel-Yagui et al., 2005; Kim et al., 2008; Roth et al., 2008; Arpel et al., 2014, 2016). Combined with molecular dynamics simulations and heteronuclear nuclear magnetic resonance (NMR) studies in membrane-mimicking environments, our results demonstrate that these IntPep are able to interact with the TM2 domain of human NEU1, disrupt mNEU1 dimerization and strongly decrease its sialidase activity at the plasma membrane.

## Materials and Methods

### Peptide Preparation

The different peptides used in molecular biology and biochemical analyses, FDPELVDPVVAAGAVVTSSGIVFFSNPAHPEFR (referred as TM2), FITC-LC-ELVDPVVAAGAVVTSSGIVFFS RKR (referred as FITC- IntPep-RKR), FITC-LC-GRKKRR QRRRPQ-AhX-PVVAAGAVVTSSGIVFFS (referred as FITC-TAT-IntPep), GRKKRRQRRRPQGGGGPVVAAGAVVTSSGI VFFS (referred as TAT-IntPep), GRKKRRQRRRPQGGGGP VVVAIAVVTSSIIAFFS (referred as TAT-mutIntPep), GRKKRR QRRRPQ (referred as TAT), ELVDPVVAAGAVVTSSGIVF FSRKR (referred as IntPep-RKR) ELVDPVVVAIAVVTSSI IAFFSRKR (referred as mutIntPep-RKR), were purchased from GeneCust Europe (Ellange, Luxembourg) and GENEPEP (Saint Jean de Védas, France). The purity of these peptides determined by RP-HPLC was about 95% according to manufacturer indications.

Peptides were solubilized in LDS micelles as described previously (Roth et al., 2008). 1 mg of each peptide was solubilized in 600 μl 2,2,2-trifluoroethanol (TFE) (Sigma). 72 μl of LDS at 1 M in phosphate buffered saline (PBS) (Sigma) and 100 μl PBS were added to the TFE-peptide mix and the mix was then vortexed. Vacuum evaporation was done using SpeedVac system (Eppendorf, Concentrator 5301) at 45°C until complete evaporation of liquid. Dried pellet was resuspended in 1 ml ultrapure water. Peptides included in micelles were incubated with cells in a small volume in order to attain a detergent concentration far less than its critical micelle concentration (CMC), permitting partitioning of the peptides in cell membranes (Bennasroune et al., 2004).

TAT-IntPep and TAT-mutIntPep were solubilized in 70% dimethyl sulfoxyde (DMSO) (Sigma) at 25 mM (stock solution). FITC-TAT-IntPep were solubilized in acetic acid 2.5% at 1 mM (stock solution).

### Dynamic Light Scattering

Micelle averaged hydrodynamic diameters (Z-ave) were determined by Dynamic Light Scattering (DLS) with a Zetasizer Nano ZS (Malvern Zetasizer Nano-ZS, Malvern Instruments, Worcestershire, United Kingdom) equipped with a laser source of wavelength of 633 nm. Each suspension was analyzed in triplicate at 25°C at a scattering angle of 173°. Water was used as a reference dispersing medium. The data were exploited with the Zetasizer Software version 7.12.

### MTT Assays

Cell viability assays were performed on COS-7 cells (ATCC^®^ CRL-1651^TM^), a fibroblast-like cell line derived from green African monkey kidney, harvested in 96 well plates at a cell density of 10,000 cells/well. After 24 h, cells were incubated with IntPep for 15 min, 30 min, 1, 24, or 48 h at a concentration range of 0.01–10 μM. Medium was removed and cells were incubated in obscurity for 4 h at 37°C with a 3-(*4-5-dimethylthiazol-2-yl*)-*2,5-diphenyltetrazolium* bromide solution (MTT, Sigma, 5 mg/ml) diluted 1/6 in PBS. After incubation, medium was removed, wells washed with PBS and 100 μl dimethyl sulfoxyde (DMSO) (Sigma) were added to each well to solubilize formazan crystals. After 5 min agitation at room temperature, cell viability was assessed at 570 nm with Infinite F200 Pro (TECAN) hardware using Magellan software.

### Cell Cultures and Transfections

Plasmid encoding human PPCA protein was provided by Pr. Alessandra d’Azzo and has been described previously (Bonten et al., 2009). Plasmid encoding human NEU1 was purchased from ImaGenes GmbH (Berlin, Germany). Plasmids encoding NEU1-Flag and NEU1-HA proteins were produced as described previously by Maurice et al. (2016). JetPEI DNA transfection reagent used for cell transfections was purchased from Polyplus transfection. COS-7 cells were harvested in 4,5 g/l glucose Dulbecco’s Modified Eagle’s Medium (DMEM) supplemented with 10% heat-inactivated fetal bovine serum, 100 units/mL penicillin, 0.1 mg/mL streptomycin at 37°C in a humidified atmosphere at 95% air and 5% CO2. For all experiments, COS-7 cells were transiently transfected with NEU1/PPCA (1:2) using JetPEI according to the manufacturer’s protocol and all experiments were performed 48 h post-transfection. THP-1 cells were harvested in RPMI 1640 medium supplemented with 10% heat-inactivated fetal bovine serum, 100 units/mL penicillin, 0.1 mg/mL streptomycin at 37°C in a humidified atmosphere at 95% air and 5% CO2. THP-1 monocytes were differentiated into adherent macrophages using 50 nM PMA (phorbol-12-myristate-13-acetate) (Calbiochem) for 72 h.

### Western Blot

Protein samples in appropriate buffers according to the experiments were diluted in Laemmli buffer (62.5 mM Tris, 2% SDS, 10% glycerol, 0.05% bromophenol blue, and pH 6.8) and heated 10 min at 100°C. After electrophoresis in a 10% acrylamide SDS-PAGE gel, proteins were transferred onto a nitrocellulose membrane at 100 V for 1 h in a Tris/glycine buffer supplemented with 10% ethanol. After blocking of the nitrocellulose membrane with 0.05% TBS Tween-20 (TBS-T) supplemented with 5% milk for 1 h at room temperature, membrane was probed with primary antibodies diluted at 1/1,000 in TBS-T with 3% BSA overnight at 4°C. Membrane was then washed in TBS-T and incubated with HRP-linked secondary antibodies diluted at 1/10,000 in TBS-T with 5% milk at room temperature. Anti-rabbit HRP-linked antibodies and anti-mouse HRP-linked antibodies (Cell Signaling) were used for protein detections. Chemiluminescent protein detection was done using ECL Prime and ODYSSEY Fc (Lycor) hardware. Rabbit monoclonal HA-Tag antibodies (C29F4) used for Western blotting were purchased from Cell Signaling.

### Immunofluorescence

COS-7 cells were grown in 24 well plates for 24 h, transfected with NEU1-Flag and PPCA encoding plasmids. 48 h after transfection, plates were washed twice with PBS and cells fixed at room temperature with 2% paraformaldehyde (PFA) (Euromedex) for 15 min. After blocking with 3% BSA (Bovine Serum Albumin) for 1 h at room temperature, coverslips were incubated with mouse monoclonal anti-Flag antibodies diluted at 1/200 in PBS with 0.3% BSA at 4°C overnight. After washes, coverslips were incubated with anti-mouse secondary antibodies coupled with AF568 (Invitrogen) diluted at 1/1,000 in PBS with 0.3% BSA for 1 h at room temperature. After washes, interfering peptides coupled with FITC (Fluorescein Isothiocyanate) were incubated with cells. After washes under agitation, coverslips were mounted in a medium containing DAPI (Prolong^®^gold, antifade, Invitrogen) and slides were visualized using confocal microscopy (Zeiss LSM 710).

### Co-immunoprecipitation

Transfected COS-7 cells grown in 10 cm Petri dishes were washed with cold PBS and resuspended in 1 mL cold TEM buffer (75 mM Tris, 2 mM EDTA, 12 mM MgCl_2_ with a protease inhibitor cocktail, 10 mM NaF, 2 mM Na_3_VO_4_, and pH 7.5). After sonication, samples were centrifuged at 600 × *g* at 4°C for 10 min to remove nuclei and non-lysed cells. Thereafter, samples were centrifuged a second time at 20,000 × *g* at 4°C for 45 min and crude membrane-containing pellets were resuspended in TEM buffer with 1% CHAPS {3-[(3-cholamidopropyl) dimethylammonio]-1-propanesulfonate} (Sigma). Peptides were incubated with cell membranes. Membranes were then solubilized for 3 h at 4°C under gentle end-over-end mixing. After a centrifugation step (20,000 × *g* for 45 min at 4°C), supernatants were recovered. Immunoprecipitation was carried out using 4 μg of mouse monoclonal anti-Flag antibodies and G-sepharose beads for 2 h at 4°C under gentle end-over-end mixing. Protein G Sepharose beads used for co-immunoprecipitation experiments were purchased from GE healthcare. Mouse monoclonal Anti-Flag^®^ M2 antibodies used for co-immunoprecipitations were purchased from Sigma. For co-immunoprecipitation experiments between NEU1-Flag and fluorescent interfering peptides, beads were washed with TEM buffer containing 1% CHAPS, resuspended in PBS and put in black 96 well plates. FITC-emitted fluorescence was detected using the SPARK 10M (TECAN) hardware (excitation: 490 nm/emission: 525 nm).

### Sialidase Activity

Transfected COS-7 cells harvested in 10 cm Petri dishes were washed with cold PBS and resuspended in 1 mL cold TEM buffer (75 mM Tris, 2 mM EDTA, 12 mM MgCl_2_ with a protease inhibitor cocktail, 10 mM NaF, 2 mM Na_3_VO_4_, pH 7.5). After sonication, samples were centrifuged at 600 × *g* (10 min, 4°C) to remove nuclei and non-lysed cells. Thereafter, samples were centrifuged at 20,000 × *g* (45 min, 4°C) and crude membrane-containing pellets were resuspended in 400 μl MES buffer (2-(*N*-morpholino) ethanesulfonic acid hydrate, 20 mM, pH 4.5) (Sigma). After quantification of proteins with a BCA protein assay (kit BCA, Pierce), sialidase activity at the plasma membrane was measured from 50 μg of crude membrane proteins. Peptides were incubated with crude membrane preparations for 15 min. Crude membrane proteins in MES buffer were then incubated with Muf-NANA at a concentration of 400 μM for 2 h at 37°C in obscurity. 2′-(4-methylumbelliferyl)-alpha-D*-N*-acetylneuraminic acid (Muf-NANA) was purchased from BioSynth. Reaction was stopped by adding Na_2_CO_3_ (Merck). Samples were then deposited in black 96 well plates and emitted florescence was measured with Infinite F200 Pro (TECAN) hardware and Magellan software (excitation: 360 nm/emission: 465 nm).

Sialidase activity at the plasma membrane of macrophages was performed as described previously (Kawecki et al., 2019). Kappa-elastin harboring the GxxPG bioactive motif was produced by chemical hydrolysis of insoluble elastin coming from bovine neck ligaments. Kappa-elastin obtained after hydrolysis was lyophilized. Differentiated THP-1 cells, seeded in 12-well culture dishes (5.10^5^ cells/well), were washed with PBS and incubated with IntPep, a reaction buffer containing 20 mM of CH_3_COONa (pH = 6.5) and 400 μM of Muf-NANA, with or without kE (50 μg/mL), for 2 h at 37°C in the dark. After incubation, the reaction was stopped by adding 0.4 M of glycine buffer (pH = 10.4) and the fluorescent 4-methylumbeliferone product released in the medium was measured using the Infinite F200 Pro (TECAN) hardware and Magellan software. NEU1 (H-300) antibodies purchased from Santa Cruz were used to assess NEU1 expression in both cell lines.

### Nuclear Magnetic Resonance Studies

The ^15^N-labeled recombinant fragment hNEU1/TMS2 fragment corresponding to the NEU1 residues R305–R341 (R^305^D VTFDPELVDPVVAAGAVVTSSGIVFFSNPAHPEFR^341^ named as hNEU1-TMS2) was produced by bacterial expression and purification as described in Maurice et al. (2016). The unlabeled recombinant peptide GSMWHHHHHHGGE LVDPVVVAIAVVTSSIIAFFSN (named as mutIntPepR), corresponding to the recombinant version of the four-point mutated peptide IntPep, was expressed in continuous exchange cell-free expression system with *Escherichia coli* S30 extract (Bocharova et al., 2016) using gene construction with GB1 as a leading fusion protein followed by thrombin cleavage site before the peptide sequence. In order to incorporate the TM fragments into membrane mimicking micelles, the peptide powders were first dissolved in 1:1 (v/v) trifluoroethanol–water mixture with the addition of dodecylphosphocholine (DPC) at detergent/peptide molar ratio (D/P) from 50:1 to 200:1 and then placed for several minutes in an ultrasound bath. The mixture was lyophilized overnight and re-dissolved at pH 6.5 in 400 μl of water buffer solution containing 20 mM sodium phosphate, 0.15 μM NaN_3_, and 5% D_2_O (v/v). In order to study homodimerization, the 0.4 mM ^15^N-labeled hNEU1-TMS2 sample having initial D/P of 50 was titrated by adding of small portions of concentrated DPC micelle suspension up to final D/P of 110. In order to study heterodimerization, the 0.3 mM ^15^N-labeled hNEU1-TMS2 sample with D/P of 120 was titrated by adding unlabeled mutIntPepR (embedded into concentrated micelle suspension with D/P of 100 of 80) up to final hNEU1-TMS2/mutIntPepR ratio (N/I) equal to 1 (with D/P of 100). NMR spectra were acquired at 313 K on 800 MHz AVANCE III spectrometer (Bruker BioSpin, Germany) equipped with triple-resonance Z-gradient cryoprobe. The ^1^H/^15^N backbone resonances and NOE connectivity pattern of hNEU1-TMS2 (0.8 mM ^15^N-labeled sample with D/P of 200) were assigned with the CARA software (Keller, 2004) using two- and three-dimensional heteronuclear NMR experiments (Cavanagh, 2007): ^1^H/^15^N-HSQC, ^1^H/^15^N-TROSY, ^15^N-edited TOCSY- and NOESY-HSQC with mixing times of 40 and 100 ms, respectively. The water-accessibility of hNEU1-TMS2 residues was analyzed by detection of chemical exchange of the amide protons with water detected by the CLEANEX experiment (Hwang et al., 1998). In order to characterize the intramolecular dynamics of hNEU1-TMS2, the effective rotation correlation times τ_*R*_ were estimated for individual amide groups of the fragments based on ^15^N CSA/dipolar cross-correlated transverse relaxation experiment acquired in interleaved fashion for the reference and attenuated spectra using a 2D ^1^H/^15^N-ct-TROSY-HSQC-based pulse sequence (Chill et al., 2006).

### Prediction of TM Helix-Helix Dimer Structures With PREDDIMER Algorithm

Preliminary 3D models of TM heterodimers were built using the PREDDIMER web-server^1^ (Polyansky et al., 2014). To exclude terminal residues from consideration, we used shortened sequence for TM2 referred to as TM2′ (ELVDPVVAAGAVVTSSGIVFFSNPA), IntPep-RKR and mutIntPep-RKR. Preliminary models were compared with each other and with the previously proposed TM2 dimer (Maurice et al., 2016).

### Simulations of Spontaneous Dimerization With the DAFT Approach

The DAFT approach (Wassenaar et al., 2015) was applied to search for possible TM2/IntPep-RKR dimeric states using 500 starting conformations, which represented non-associated states with two helical monomers in 1-palmitoyl-2-oleoyl-phosphatidylcholine (POPC) membrane separated by 2–3 nm distance. After spontaneous dimerization during 500 ns molecular dynamics (MD) simulation all stable complexes were selected and clustered based on the energy of helix-helix interaction estimates and the peptide orientation with respect to each other. Structure of the TM2/mutIntPep-RKR complex corresponds to the representative model derived from the largest cluster of the resulting TM2/IntPep-RKR dimers. Four point mutations were further made using the Pymol software (DeLano, 2002).

### Molecular Modeling

Monomers of each peptide (TM2, TM2′, TAT-IntPep, TAT-mutIntPep, IntPep-RKR, and mutIntPep-RKR) were built in an ideal α-helical conformation with the Pymol software (DeLano, 2002). All peptides were aligned along the membrane normal and embedded into a 1-palmitoyl-2-oleoyl-phosphatidylcholine (POPC) lipid bilayer, hydrated with TIP3P water molecules (Jorgensen et al., 1983) and added ions (150 mM KCl). This was done using the CHARMM-GUI (Jo et al., 2008). The systems were simulated according to the protocol described below.

The full system was minimized and equilibrated for 500 ps at constant volume and temperature (310 K) using the Berendsen thermostat (Hwang et al., 1998), with protein heavy atoms fixed. The system was further simulated for 10 ns under constant pressure and constant temperature (310 K), with protein heavy atoms fixed. The resulting system was used as a starting point for productive MD runs. All simulations were performed using the Gromacs software (Pronk et al., 2013), the AMBER 14SB force field for proteins (Lindorff-Larsen et al., 2010), LIPIDS force field for lipids (Dickson et al., 2014), and TIP3P water (Jorgensen et al., 1983). The calculations were performed at constant temperature (310 K) using V-rescale thermostat (Bussi et al., 2007) and Parrinello–Rahman pressure (one bar) coupling algorithm (Bussi et al., 2007). The integration time step was 2 fs and all bonds were constrained using P-LINCS (Hess, 2008). Water molecules were kept rigid using the SETTLE algorithm. Lennard–Jones interactions were cutted at 1.2 nm. Long-range electrostatic interactions were treated using the particle mesh Ewald approach (Cheatham et al., 2002) with a 1.2 nm direct space cutoff. The neighbor list was updated every 10 ps and the center-of-mass motion of the entire system was removed at every step. For each system, MD simulations of 250 ns were carried out. Analyses were conducted on the last 100 ns to discard the equilibration time in all cases. At least three replicas were performed for each system.

Simulation analyses were carried out using the MD analysis (Michaud-Agrawal et al., 2011) library with our home-made scripts in Python.

### Estimation of the Dimer Stability

The most representative conformations of TM2/IntPep-RKR and TM2/mutIntPep-RKR heterodimers were extracted from DAFT results, converted to all-atom models and embedded into hydrated POPC bilayer. Several selected models generated by PREDDIMER were also subjected to MD simulations in explicit POPC bilayer in order to estimate their structural stability.

Secondary structure and root-mean-square deviation (RMSD) of backbone atoms coordinates were controlled during the simulations to ensure structure stability. Dimer geometry was described in terms of helices tilt with respect to the membrane normal and crossing angle between the two helices axes. Intermonomer contacts were determined by calculation of per-residue solvent-exposed surface. Finally, representative stable dimer structures were extracted by cluster analysis and used in the free energy calculations.

### Free Energy Calculations

To estimate the free energy of dimerization of TM peptides (TM2′, IntPep-RKR, mutIntPep-RKR), umbrella sampling approach with harmonic restraining potential was used. Homo- and heterodimers were considered. Starting TM2′-TM2′ dimer conformation was taken from our previous work (Maurice et al., 2016), TM2′-IntPep-RKR and TM2′-mutIntPep-RKR heterodimers were selected as representative stable states from MD simulations of the structures of TM2′-TM2′ dimer generated by PREDDIMER. Here, we switched to faster united-atom Gromos 43a2 forcefield with extended Berger lipids (Berger et al., 1997) and SPC water (Berendsen et al., 1984). Short 30 ns MD trajectories were calculated to ensure that change of the forcefield does not affect the dimer structures (RMSD < 0.05 nm). Then, the distance between monomers’ centers of mass was selected as a reaction coordinate varying from 0.75 to 2.20 nm with a step 0.05 nm, thus resulting in 32 simulation windows. Starting conformations for each window were constructed by translation of the monomers in the membrane plane along the reaction coordinate, followed by 30 ns of MD simulation with restrained protein segments during equilibration of the system. To calculate energy profiles, additional 50-ns productive MD run was executed for each window. Free energy profiles were obtained using the weighted histogram analysis method (WHAM) and “Bayesian bootstrap” analysis to estimate statistical errors (implemented in the Gromacs package) (Hub et al., 2010). The values of dimerization free energy were determined as the depth of the profile minima.

### Statistical Analyses

Results are expressed as mean ± SEM. Statistical significance was evaluated using Student’s *t* test or ANOVA followed by a Dunnett’s multiple comparison test.

## Results

In this study, two approaches were used in order to deliver the IntPep targeting the TM2 domain of human mNEU1 into cells: (i) in the first one (“TAT approach”), IntPep was linked to the TAT peptide sequence, rich in lysine and arginine residues; (ii) in the second method (“LDS approach”), IntPep was solubilized into positively charged LDS micelles. Both approaches are known as effective strategies to deliver cargoes into cells (Arpel et al., 2014, 2016). For the TAT approach, the GRKKRRQRRRPQ-GGGG-P_316_VVAAGAVVTSSGIVFFS_333_ (TAT-IntPep) and the mutated GRKKRRQRRRPQ-GGGG-P_316_VVVAIAVVTSSIIAFFS_333_ (TAT-mutIntPep) sequences were used. A poly-gly linker (GGGG) between the TAT sequence and the IntPep was introduced to allow flexibility of the construct. For the LDS approach, sequences of the two interfering peptides used were E_312_LVDPVVAAGAVVTSSGIVFFS_333_-RKR (IntPep-RKR) and E_312_LVDPVVVAIAVVTSSIIAFFS_333_-RKR (mutant variant, mutIntPep-RKR). For this second approach, RKR motif was added at the C-terminus of both IntPep. This motif is known to optimize the orientation of peptides when they are inserted into the membranes (Roth et al., 2008). The length of the interfering sequence was also increased at the N-terminus (E_312_LVD_315_). The mutated sequences were chosen based on previous works showing that the following four residues are critical for mNEU1 dimerization and sialidase activity: A_319_, G_321_, G_328_, and V_330_ (Maurice et al., 2016). In some experiments, a FITC probe was added at the N-terminus of IntPep both to localize the interfering peptides in cells and assess their ability to interact with mNEU1.

For each peptide delivery approach, experiments were first conduced to characterize the IntPep (stability of their secondary structure in monomer by MD simulations, homogeneity of the micellar preparations by Dynamic Light Scattering, effects of both IntPep formulations on cell viability by MTT assays). The interactions between IntPep and NEU1 TM2 domain or mNEU1 were next studied by NMR or confocal laser scanning microscopy and co-immunoprecipitation experiments, respectively. Then, effects of IntPep on the formation of mNEU1 dimers and sialidase activity were evaluated in biochemical essays. Finally, the most probable 3D models of TM2/IntPep dimers were elaborated using two independent computational approaches and the structural stability of the resulting consensus models was explored via the MD simulations in lipid bilayer. Based on the totality of the obtained experimental and *in silico* structural data, the strength of TM helix-helix association (expressed in terms of the free energy of their interaction) was calculated, thus providing a direct way to interpretation of molecular aspects of IntPep action on NEU1 dimerization in TM domain and its activity.

### Structural Characterization of the Interfering Peptides

For each monomer, stability of its secondary structure was evaluated in the course of a 250 ns MD simulation. For TM2, TAT-IntPep and IntPep-RKR simulations, the central part of the peptides defined between P_316_ and S_333_ fits well with an α-helical structure and only extremities of the peptides, in contact with water, have no specific secondary structure (Supplementary Figures 1A–C). Same results were obtained for their monomeric mutant counterparts (Supplementary Figures 1D,E). During all the simulation time, amino acid residues, which are embedded into the POPC bilayer, revealed the α-helical structure. For TAT-IntPep and TAT-mutIntPep, α-helices immersed into the lipid bilayer are defined between the last glycine of the linker and A_330_.

We then focus on the transmembrane α-helix stability and orientation in lipid bilayers. The structural stability of each system was evaluated in terms of the root-mean-square deviation (RMSD) of coordinates of C_α_ atoms of the TM helix (Cα-RMSD_helix_). For monomers, the Cα-RMSD_helix_ values all along the simulation time range from 0.06 ± 0.01 (for IntPep-RKR) to 0.08 ± 0.01 nm (for mutIntPep-RKR) indicating that no major structural reorganization occurred. The same behavior was observed for TM2 that shows small and stable Cα-RMSD_helix_ of 0.07 ± 0.01 nm. For TAT-IntPep (TAT-IntPep and TAT-mutIntPep), more important drifts ranging from 0.27 ± 0.03 (TAT-IntPep) to 0.28 ± 0.02 nm (TAT-mutIntPep) were observed compared to the initial structures.

To control the orientation of peptides with respect to the lipid bilayer, the angle formed between the helix axis of the peptide and the normal to the POPC membrane, called the tilt angle, was evaluated (Ozdirekcan et al., 2007). Indeed, according to the lipid composition, each TM α-helix has a specific orientation. The tilt angle depends of the α-helix length and the hydrophobic mismatch effects (Holt and Killian, 2010). When embedded into the bilayer, IntPep-RKR and mutIntPep-RKR reveal comparable tilt angles of 13 ± 6° and 16 ± 6°, respectively. Quite similar tilt angles of 20 ± 6° and 18 ± 7° were also observed for TAT-IntPep and TAT-mutIntPep, respectively. Moreover, TM2 inserted into the lipid bilayer demonstrates a tilt angle of 27 ± 8°. Altogether, the data underline the structural stability of all monomers embedded into POPC bilayers all along the simulation time regarding their α-helix secondary structure and small Cα-RMSD_helix_ values. For each strategy (TAT or LDS), the tilt angles are similar between the wild type and the mutant forms of IntPep. Thus, the introduction of the four point mutations has no impact on the overall structural and dynamic properties of the membrane-bound IntPep monomers.

Dynamic Light Scattering experiments were also performed to determine the size of empty micelles and those containing IntPep-RKR, mutIntPep-RKR, and FITC-IntPep-RKR peptides. A similar size of around 2 nm was detected showing that the different micellar preparations are homogeneous in size (data not shown).

We finally evaluated the effects on both IntPep formulations on cell viability in COS-7 cells (Supplementary Figures 2A,B). After 24 h incubation with both TAT and TAT-IntPep for concentrations varying from 1 to 10 μM, no cellular toxicity was observed (Supplementary Figure 2A). Similar results were observed with empty LDS micelles, IntPep-RKR, and mutIntPep-RKR micelles at concentration of 0.1 μM. However, a cellular toxicity increasing with LDS concentration was detected for peptide concentrations starting at 1 μM (Supplementary Figure 2B). Therefore, we decided to use TAT peptides at concentrations varying from 1 to 10 μM and LDS micelles containing RKR peptides at concentration of 0.1 μM.

### Interaction Between Interfering Peptides and mNEU1

**FIGURE 1:**
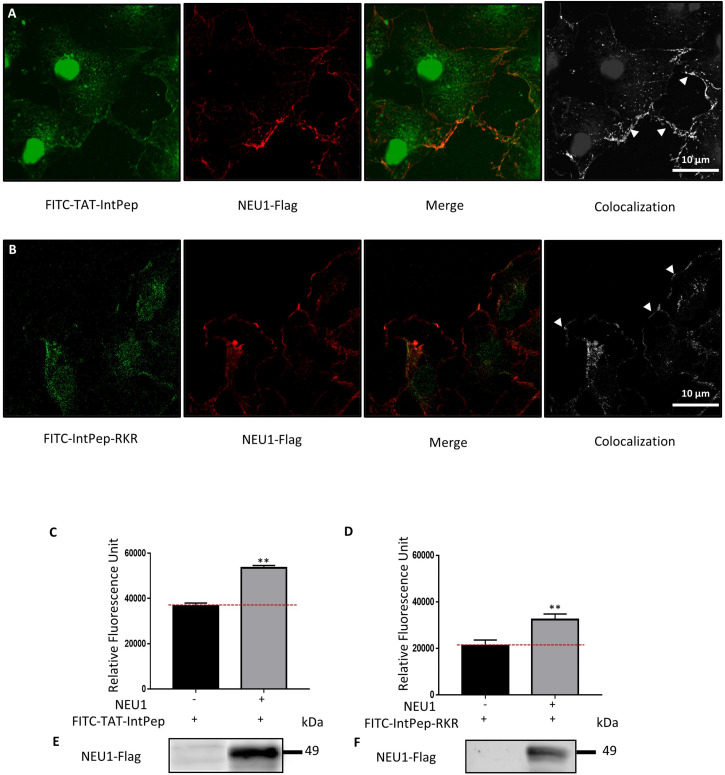
Interaction between interfering peptides and membrane NEU1. Localization of FITC-interfering peptides in COS-7 cells overexpressing NEU1. **(A)** Localization of FITC-TAT-IntPep used at 10 μM (green) and NEU1-Flag (red). Colocalization areas are represented in white (right). **(B)** Localization of FITC-IntPep-RKR used at 0.1 μM (green) and NEU1-Flag (red). Colocalization areas are represented in white (right). **(C–F)** Fluorescence quantification of FITC-interfering peptides after immunoprecipitation of NEU1-Flag. COS-7 cells were transfected by plasmids encoding NEU1-Flag and PPCA proteins and incubated with FITC-TAT-IntPep at 5 μM **(C)** (*n* = 3) or FITC-IntPep-RKR at 0.1 μM **(D)** (*n* = 4). **(E,F)** NEU1-Flag expression in untransfected COS-7 cells (left) and transfected COS-7 cells (right) for FITC-TAT-IntPep **(E)** and FITC-IntPep-RKR **(F)**. **p < 0.01, *t* test.

The ability of IntPep to colocalize with their target at the plasma membrane of cells was studied by confocal microscopy. FITC-labeled IntPep were therefore used and incubated for 60 min with adherent COS-7 cells overexpressing NEU1-Flag. For both delivering strategies, colocalization areas between fluorescent IntPep and NEU1-Flag were clearly observed at the plasma membrane of COS-7 cells (Figures 1A,B). Moreover, a nuclear staining was observed with the TAT strategy. Interaction of IntPep with mNEU1 was further assessed by co-immunoprecipitation experiments from crude membrane preparations. NEU1-flag was immunoprecipitated and the amount of co-immunoprecipitated FITC-IntPep was evaluated. Fluorescence was significantly increased by 46% for the TAT approach and by 53% for the RKR approach in cells overexpressing NEU1 compared to untransfected COS-7 cells (Figures 1C–F). Taken together, both confocal microscopy and co-immunoprecipitation experiments show that IntPep are able to colocalize and interact with their target mNEU1.

### Effects of Interfering Peptides on the Formation of mNEU1 Dimers

**FIGURE 2:**
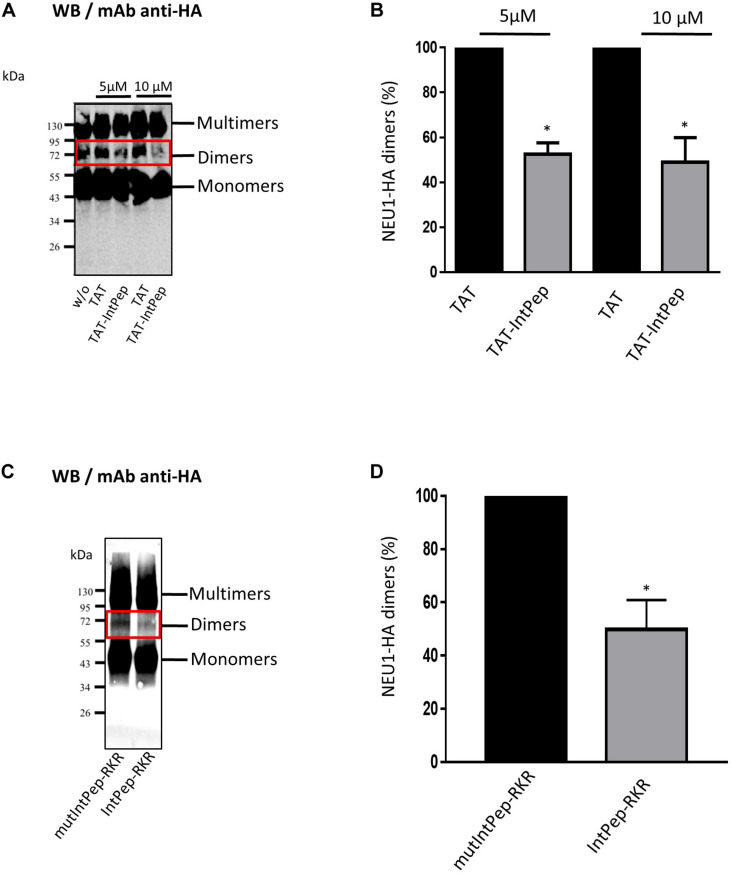
Effects of interfering peptides on NEU1 dimerization. COS-7 cells were transfected by plasmids encoding NEU1 and PPCA proteins and incubated with the different IntPep during membrane protein solubilization. **(A)** Western blot showing the effects of TAT and TAT-IntPep on NEU1 dimerization at 5 or 10 μM. Rabbit monoclonal HA-Tag antibodies were used for Western blotting. **(B)** Quantification of the dimers formed in presence of TAT or TAT-InpPep at 5 or 10 μM (*n* = 4). **(C)** Western blot showing the effects of mutIntPep-RKR and IntPep-RKR on NEU1 dimerization at 0.1 μM. Rabbit monoclonal HA-Tag antibodies were used for Western blotting. **(D)** Quantification of the dimers in presence of mutIntPep-RKR or IntPep-RKR at 0.1 μM (*n* = 4) (w/o: without peptide) (*p < 0.05, *t* test).

After pointing out the interaction between IntPep and their target mNEU1, the ability of IntPep to inhibit homodimerization of mNEU1 was studied. As previously shown (Maurice et al., 2016), NEU1 can be observed in monomeric, dimeric or multimeric forms on migration profiles obtained from crude membrane preparations. NEU1 dimers were identified by the presence of bands of 70–72 kDa on Western blots (Figures 2A,C). The formation of NEU1 dimers was decreased in the presence of TAT-IntPep compared to TAT alone. A significant decrease of 47 ± 5% of NEU1 dimers was observed with TAT-IntPep in comparison with TAT peptides at 5 μM. Similarly, a decrease of 51 ± 11% was detected with TAT-IntPep in comparison with TAT peptides at 10 μM (Figures 2A,B). Similar effects were obtained for IntPep-RKR compared to mutIntPep-RKR. A significant decrease of 50 ± 11% of NEU1 dimers was observed with IntPep-RKR in comparison with its mutant counterpart at 0.1 μM (Figures 2C,D). Altogether, our results show that IntPep are able to decrease mNEU1 dimerization when using both delivery approaches.

### Effects of Interfering Peptides on mNEU1 Sialidase Activity

**FIGURE 3:**
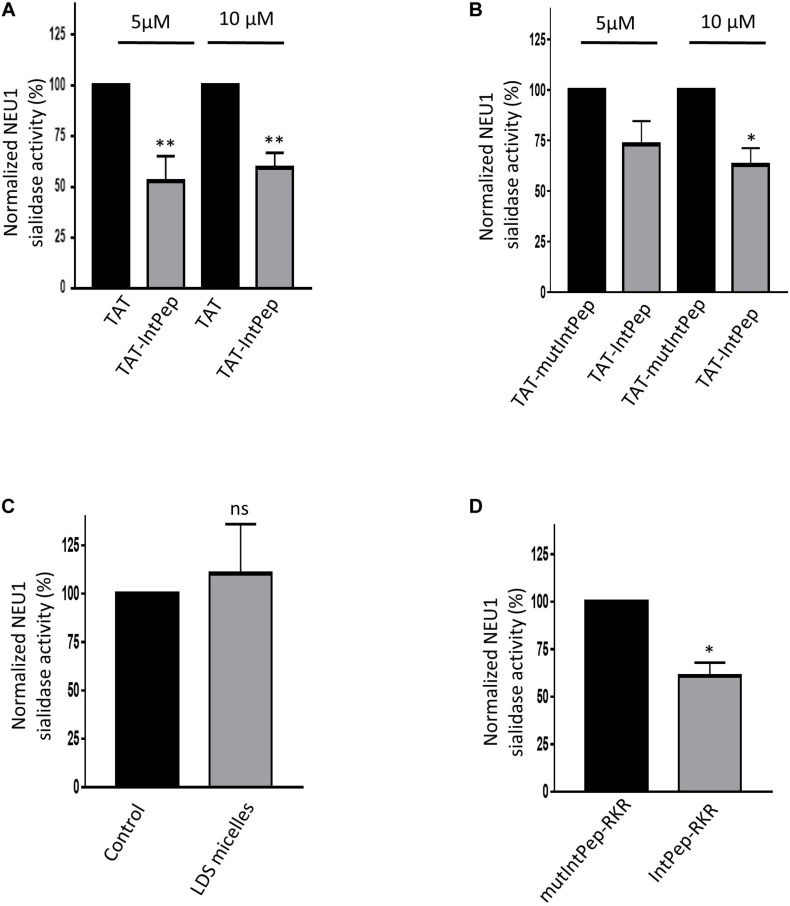
Effects of interfering peptides on membrane NEU1 sialidase activity in NEU1 overexpressing cells. Effects of TAT-IntPep on sialidase activity in COS-7 cells. 48 h post-transfection, membrane preparations of COS-7 cells were incubated with TAT-IntPep, TAT-mutIntPep or TAT. **(A)** Normalized sialidase activity after 15 min incubation with TAT or TAT-IntPep at 5 or 10 μM. Results are represented compared to the TAT peptide condition normalized to 100% (*n* = 7). **(B)** Normalized NEU1 sialidase activity after 15 min incubation with TAT-mutIntPep or TAT-IntPep at 5 μM (*n* = 3) or 10 μM (*n* = 4). Results are represented compared to the TAT-mutIntPep condition normalized to 100%. Effects of IntPep-RKR on sialidase activity in COS-7 cells. **(C)** Normalized sialidase activity after 15 min incubation without (Control) or with empty micelles only. Results are represented compared to the no peptide control condition normalized to 100% (*n* = 3). **(D)** Normalized NEU1 sialidase activity after 15 min incubation with IntPep-RKR or with mutIntPep-RKR at 0.1 μm. Results are represented compared to the mutIntPep-RKR control condition normalized to 100% (*n* = 3) (*p < 0.05, **p < 0.01, ns: non-significant, *t* test).

We next evaluated the ability of IntPep to decrease mNEU1 sialidase activity in crude membrane preparations of COS-7 cells overexpressing NEU1. A fourfold increase of the sialidase activity was observed between untransfected cells and COS-7 cells transfected by plasmids encoding for NEU1 (not shown). The effects of IntPep were then assessed after 15 min incubation. Significant decrease of mNEU1 sialidase activity of 47 ± 12% and 41 ± 8% was observed with TAT-IntPep compared to TAT peptides at 5 and 10 μM, respectively (Figure 3A). Similarly, a significant decrease of mNEU1 sialidase activity of 37 ± 8% was registered with TAT-IntPep compared to TAT-mutPepInt at 10 μM (Figure 3B). Moreover, a decrease of mNEU1 sialidase activity of 39 ± 7% was detected for IntPep-RKR with respect to mutIntPep-RKR at 0.1 μM (Figure 3D). No difference in sialidase activities between the control condition (no peptide and no micelle) and the empty LDS micelle condition was noticed (Figure 3C). In both strategies, IntPep are able to decrease sialidase activity of the mNEU1 in NEU1 overexpressing cells.

**FIGURE 4:**
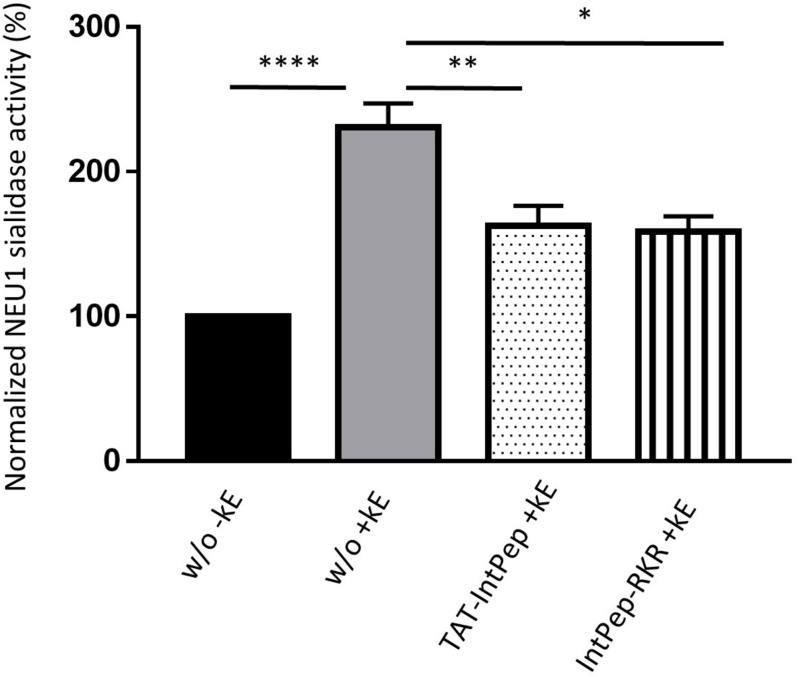
Effects of interfering peptides on membrane NEU1 sialidase activity in adherent macrophages under κ-elastin stimulation. Sialidase activity was triggered (+ kE) or not (-kE) by κ-elastin in adherent macrophages. Adherent macrophages were incubated with TAT-IntPep, IntPep-RKR or without peptide (w/o) for 120 min and sialidase activity was measured on adherent cells (*n* = 3–9). *p < 0.05, **p < 0.01, ****p < 0.0001, and *t* test.

As previously reported, membrane sialidase activity triggered by κ-elastin stimulation of THP1-derived macrophages is dependent of NEU1 (Kawecki et al., 2019). Therefore, the effects of IntPep in this cell model, which endogenously expressed NEU1, were evaluated. After κ-elastin stimulation, mNEU1 sialidase activity of macrophages increases compared to non-stimulated cells (231 ± 16% vs 100%). In the presence of TAT-IntPep, the sialidase activity of NEU1 decreased compared to untreated stimulated cells (162 ± 14% vs 231 ± 16%). In the presence of IntPep-RKR, the activity decreased in comparison to untreated stimulated cells (159 ± 10% vs 231 ± 16%) (Figure 4). These results therefore show the ability of IntPep to block membrane sialidase activity triggered by κ-elastin in THP1-derived macrophages.

To reveal the molecular mechanisms underlying the above observations on the effect of IntPep on NEU1 dimerization and activity, a combined experimental/computational structural biophysics approach was developed and applied to all the peptides under study. It consists in the following stages: (i) Computational prediction of the most probable dimeric models; (ii) Evaluation of their structural parameters in lipid membrane using NMR spectroscopy in micelles and MD simulations; (iii) Selection of the best consensus models from the generated ensemble of structures; (iv) Calculation of the free energy of helix-helix association in explicit membrane.

### Possible Heterodimer Structures Revealed by PREDDIMER and DAFT Approaches

In total, 8 and 7 dimeric structures were generated for TM2′/IntPep-RKR and TM2′/mutIntPep-RKR, respectively. The first two structures in each set represented parallel and crossed helix packing having highest values of the scoring function: 2.1 and 1.9 for parallel (−15°) and crossed (+ 60°) TM2′/IntPep-RKR heterodimers, respectively; 2.6 and 2.2 for crossed (+45°) and parallel (+5°) TM2′/mutIntPep-RKR dimers. However, the found crossed states have dimerization interfaces on the opposite sides of TM2′ helix, while the parallel ones are very similar (Cα-RMSD value is below 0.2 nm), and close to the structure proposed earlier (Cα-RMSD is 0.12 and 0.22 nm for heterodimers involving IntPep and mutIntPep, respectively) (Maurice et al., 2016). Other structures proposed by the algorithm have lower scoring values, and can be treated as derived from these two structures, as they have similar interface, but varying crossing angle.

When using coarse-grained DAFT approach, we observed dimer formation in 466 of 500 runs. As we have a large set of varying structures (including non-symmetrical ones), we used clustering to reduce the set and select the representative structures. Here, we found two major clusters, and the largest one represents a right-handed dimer (crossing angle is −50°). It has the same residues on the dimerization interface, as in the parallel structure proposed by PREDDIMER, so we also took it into consideration.

### Stability of the Dimers Formed by Interfering Peptides With TM2

**FIGURE 5:**
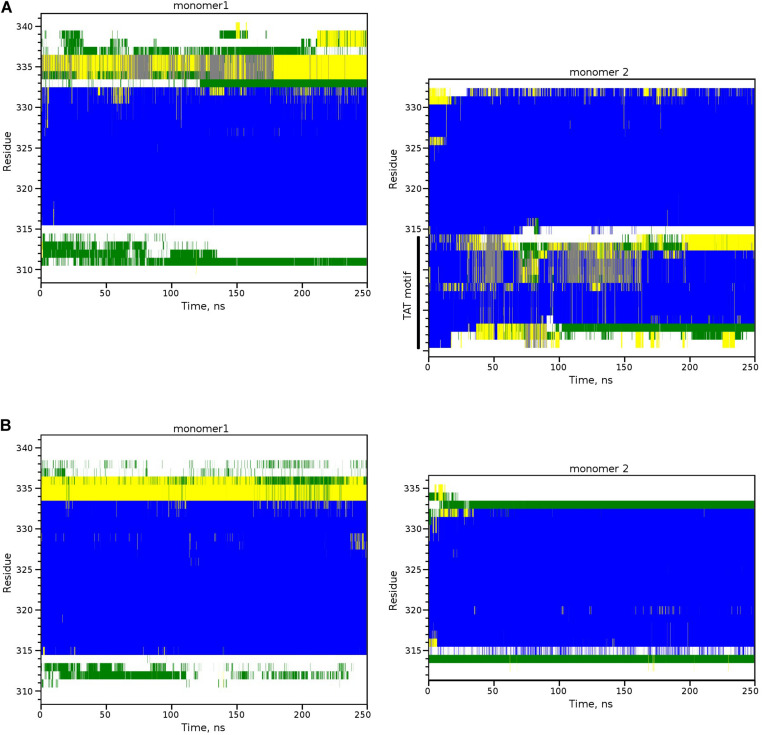
Secondary structure of TM2-interfering peptide heterodimers inside the POPC membrane. Secondary structure evolution for two monomers of the TM2/TAT-IntPep dimer **(A)** and the TM2/IntPep-RKR dimer **(B)**. α-helical structure is in blue, turn and bend regions are in green and yellow, 3_10_-helix is in gray, and unstructured fragments are in white. Residue numbering corresponding to NEU1 is shown on the left.

First, using both modifications of IntPep (TAT and RKR), TM2/IntPep dimers preserve stable α-helical structure all along the simulation time indicating that the dimer formation does not perturb the secondary structure (Figures 5A,B). In these simulations, Cα-RMSD_helix_ values are bigger for IntPep-RKR and mutIntPep-RKR in the dimer than in the corresponding monomers: 0.06 ± 0.01 nm and 0.08 ± 0.01 nm in monomer and 0.13 ± 0.03 nm in both dimers with TM2. In contrast, a decrease of Cα-RMSD_helix_ for TAT-IntPep and TAT-mutIntPep in dimer is observed compared with their monomeric forms. The corresponding RMSD values for TAT-IntPep decrease from 0.27 ± 0.03 in monomer to 0.17 ± 0.02 nm in dimer and for TAT-mutIntPep, from 0.28 ± 0.02 in monomer to 0.14 ± 0.01 nm in dimer. This difference of behavior of Cα-RMSD_helix_ between the TAT and RKR approaches may come from interaction of the TAT flag with the polar heads of lipids. Low (0.1–0.3 nm) values of Cα-RMSD_helix_ in the dimer prove stability of the dimer. The difference of Cα-RMSD_helix_ values between the monomers and the dimers underlines changes of each monomer orientation to find the best complementary interface upon its binding to the target TM2.

**FIGURE 6:**
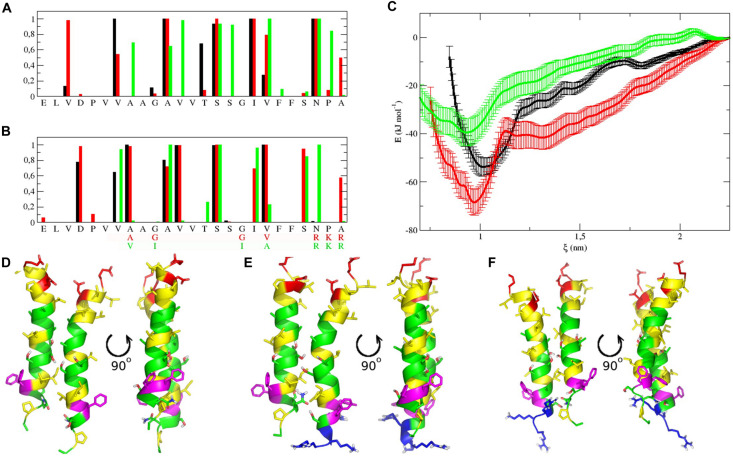
Dimerization interfaces, free energy profiles calculated for dimers in POPC membrane, and representative structures. **(A,B)** Contact probability calculated from MD simulations for TM2′/TM2′ homodimer (black), TM2′/IntPep-RKR (red) and TM2′-mutIntPep-RKR (green) heterodimers. First monomer on plot **(A)** is TM2′ in all cases, while the second one is replaced by interfering peptides on plot **(B)**. **(C)** Free energy change with the distance between monomers in TM2′/TM2′ homodimer (black), TM2′/IntPep-RKR heterodimer (red) and TM2′/mutIntPep-RKR heterodimer (green). **(D–F)** Front and side views of representative dimer structures corresponding to free energy minima for TM2′/TM2′ homodimer, TM2′/IntPep-RKR and TM2′/mutIntPep-RKR heterodimers, respectively. Polar residues are in green, positively and negatively charged in blue and red, aliphatic are in yellow, and aromatic are shown in violet. N-terminus is pointing up.

For heterodimers with more parallel packing, we observed very similar RMSD values (calculated for central parts of both monomers, residues Val317-Ser333): 0.13 ± 0.03 and 0.14 ± 0.02 nm for TM2′/IntPep-RKR and TM2′/mutIntPep-RKR, respectively. The difference between the TM2′/TM2′ homodimer (Maurice et al., 2016) and these heterodimers was also estimated by RMSD in a similar way resulting in 0.28 and 0.31 nm difference after MD (0.12 and 0.22 nm before MD) for TM2′/IntPep-RKR and TM2′/mutIntPep-RKR, respectively. These values indicate quite a high degree of similarity between the structures having similar packing interface, but different crossing angle: after MD we observed right-handed dimers for TM2′/TM2′ (−15 ± 3°) and TM2′/mutIntPep-RKR (−30 ± 3°), as well as parallel dimer TM2′/IntPep-RKR (0 ± 5°). Probably, the shorter sequence of TM2′ peptide allows almost parallel packing with increased number of intermonomer contacts (Figures 6A,B).

### Structural Characterization of TM2/IntPep Dimers With MD Simulations

We showed that for both delivering strategies, the IntPep are localized in cell membrane in close proximity with NEU1 and are able to inhibit NEU1 dimerization and its sialidase activity. Based on these results, it is strongly suggested that IntPep directly interact with NEU1 TM2 domain and disturb its orientation within the membrane. The stability of helical conformations for monomers in membrane, their orientation with respect to the membrane, and the impact of IntPep on the orientation of NEU1 TM2 dimers within the membrane were then evaluated using molecular dynamics simulation approaches at the atomic scale. First, we performed calculation of the tilt angles for each monomer in all possible dimer configurations. It was shown that dimerization with any partners didn’t strongly change overall orientation of NEU1 TM2 helix itself (average values in monomer and dimer were 27 ± 8° and 21 ± 6°, respectively). Regarding the helix reorientation from the monomer to the dimer state, the tilt angle of IntPep-RKR, is almost twofold higher (from 13 ± 6° in monomer to 23 ± 4° when dimerized with TM2) showing important reorientation of IntPep-RKR embedded in the bilayer. Similar observation was made for the mutIntPep-RKR (16 ± 6° and 31 ± 4°, respectively). For TAT-IntPep, the tilt angle increased to a lesser extent than for IntPep-RKR—from 20 ± 6° in monomer to 25 ± 6° in the dimer. No difference was observed between the TAT-mutIntPep in monomer and dimer: 18 ± 7° and 17 ± 6°, respectively. So, both RKR-peptides seriously reorient when adapting to TM2. Changes of helix orientation with respect to the membrane for TAT-derivatives were less obvious due to the strong interaction of the polar TAT-motif with lipid head groups (Herce and Garcia, 2007; Yesylevskyy et al., 2009) which reduces transmembrane helix mobility.

To further characterize the interactions between TM helices, the crossing angle (θ) between the two α-helix axes was also monitored. In all simulations of the dimers, the angle corresponds to a right-handed or parallel structure of the dimer, which is rather common for transmembrane homodimers (MacKenzie, 1997; Bocharov et al., 2008; Mineev et al., 2010). When using longer TM2 sequence, TM2/IntPep-RKR, and TM2/mutIntPep-RKR dimers reveal crossing angles of −56 ± 5° and −48 ± 7°, respectively. In the case of TM2/TAT-IntPep and TM2/TAT-mutIntPep dimers, the values of θ were −56 ± 9° and −30 ± 7°, respectively. However, with shorter TM2′ fragment, a more parallel orientation of monomers was observed: with θ values of −15 ± 3° in TM2′/TM2′, 0 ± 6° in TM2′/IntPep-RKR, and −29 ± 3° in TM2′/mutIntPep-RKR. Interestingly, such a wide distribution of θ values corresponds to the same interaction interface with A/V_319_, A_321_, V_322_, S_326_, and V/A_330_ residues forming its “core.” Introduction of the four point mutations within the IntPep results in less variable θ angle as compared to the wild type dimer.

Taken together, these MD data using both TAT- and RKR-strategies strongly suggest that IntPep mimicking the TM2 domain of NEU1 are more structurally adaptive upon binding to TM2 than their mutant counterparts. Although the obtained geometric characteristics of the considered dimeric states do not directly explain the effects of peptides on dimerization and activity of NEU1 observed in experiments, these parameters of TM helix packing confirmed the conformational stability in the membrane of the resulting dimer models and demonstrated the role of the introduced mutations in such helix-helix complexes. In addition, it is important to note that based on the entire set of MD data, only two groups of dimer models – right-handed and parallel – with similar interfaces were identified. The answer to the question of which model is preferable for calculating the strength of helix-helix association, was received from the NMR experiments in a membrane-mimic medium. These are described below.

### Transmembrane Segment Dimerization Study by NMR Spectroscopy

**FIGURE 7:**
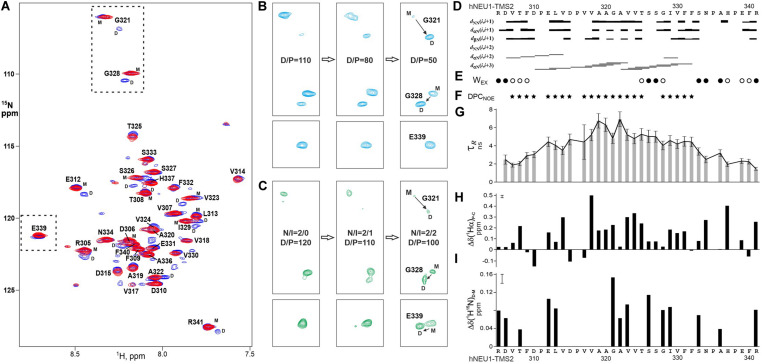
Homo- and heterodimerizations of hNEU1 TM2 segment revealed by NMR studies in membrane-mimicking environment. **(A)** Overlaid 1H/15N-TROSY spectra of 15N-labeled hNEU1-TMS2 embedded into DPC micelles at the detergent/protein molar ratio (D/P) of 100 (in blue) and 200 (in red), corresponding to the monomer-dimer transitions and mostly monomeric state of the TM segment, respectively, are shown. The 1H-15N backbone resonance assignments are marked. **(B)** Sequential fragments of 1H/15N-TROSY spectra present the G321/G328 and E339amide group cross-peaks of monomeric (M) and homodimeric (D) states of 15N-labeled NEU1-TMS2 in dependence of D/P varied from 110 to 50. **(C)** Sequential fragments of 1H/15N-TROSY spectra present the G321/G328 and E339amide group cross-peaks of monomeric (M) and heterodimeric (D) states of 15N-labeled hNEU1-TMS2 in dependence of hNEU1-TMS2/mutIntPepR ratio (N/I). **(D)** The pattern of intramolecular nuclear Overhauser effect (NOE) connectivities (of sequential and medium ranges) observed between protons of hNEU1-TMS2 in 3D 15N-edited NOESY experiment. Medium range NOE connectivities shown by horizontal lines *d*α*N(i,i* + *3)* indicate an distinct α-helical structure of the hydrophobic span V317–F332 of hNEU1-TMS2 span embedded into the micelle, while region D^310^–P^316^ has nascent helical structure. **(E)** Water accessibility (Wex) to the hNEU1-TMS2 amide groups was detected by appearance of weak (open circles) or strong (solid circles) cross peaks in the CLEANEX and 15N-edited NOESY (on the water frequency) spectra resulting from direct NOE, exchange-relayed NOE or chemical exchange of the amide protons. Wex distribution pattern indicates an incorporation of the hNEU1-TMS2 span E312–F332 into the micelle. **(F)** hNEU1-TMS2 residues having intermolecular NOE contacts between their amides and CH2 groups of DPC in 15N-edited NOESY spectrum (on ∼1.35 ppm) are marked by solid stars. The protein-lipid contact pattern reveals a penetration of the hNEU1-TMS2 span across the micelle hydrophobic core as well as some submerging of the N-terminal juxtamembrane region into micelle surface. **(G)** Local rotation correlation times *τ*R estimated from 15N CSA/dipolar cross-correlated transverse relaxation of the hNEU1-TMS2 amide groups are shown. Smaller relaxation times indicate relatively higher flexibility of the N- and C-terminal regions surrounding the span E312–F332. **(H)** Secondary 1Hα chemical shifts Δδ(1Hα)P-C represent the difference between the measured chemical shift and a typical random-coil chemical shift. Pronounced positive secondary 1Hα chemical shifts indicate a helical structure conformation of the protein (Cavanagh, 2007). **(I)** Generalized chemical shift changes Δδ(1H15N)d-m were calculated as the geometrical distance (with weighting of 1H shifts by a factor of 5 compared to 15N shifts) between the amide cross-peaks assigned to the dimeric and monomeric hNEU1-TMS2 states in the 1H/15N-TROSY spectra. The measurement uncertainty is shown in the upper left corner.

In order to detect the homodimerization of the hNEU1 TM2 segment, the ^15^N-labeled recombinant fragment hNEU1-TMS2, which included TM segment R^305^–R^341^, was prepared in the aqueous suspension of DPC at detergent/peptide molar ratio (D/P) varied from 50 to 200 (the first corresponding to one peptide embedded into one micelle in average). The patterns of intra- and intermolecular NOE connectivities (Figures 7D,F), secondary ^1^Hα chemical shifts (Figure 7H), water accessibility to the amide groups (Figure 7E) and local rotation correlation times τ_*R*_ (Figure 7G) together reveal that the hNEU1-TMS2 span E^312^–F^332^ incorporates into micelle with distinct α-helical structure from V^317^ to F^332^ and apparently nascent helical structure from D^310^ to P^316^, whereas the flanking N- and C-terminal juxtamembrane regions have nearly unrestricted mobility. Observed D/P-dependent signal doubling in the NMR spectra (Figures 7A,B,I) suggests that hNEU1-TMS2 participates in slow monomer-dimer transitions environment typical to the “weak” association (Bocharov et al., 2012, 2016) of TM helices in the membrane mimicking environments. The signal doubling of cross peaks of G^321^–G^328^ amide groups indicates the TM2 self-association through an elongated dimerization interface composed of the residues with small side-chains from two relatively polar motifs AAGA^322^ and TSSG^328^, implying a small crossing angle between the helical subunits in the TM dimer. The heterodimerization of hNEU1-TMS2 with the peptide mutIntPepR, including the four-point mutated TM2 segment E^309^ – N^334^, was monitored by variation of hNEU1-TMS2/mutIntPepR (N/I) molar ratio in the micellar environment. Stepwise addition of mutIntPepR to the hNEU1-TMS2 sample at ratio D/P maintained near 100 results in protein heterodimerization revealed by pronounced NMR signal doubling at N/I equal to 1 (Figure 7C). Decreasing N/I leads to oligomerization and further precipitation of the peptides. As for the mutant counterparts, addition of IntPepR resulted in precipitation of the hNEU1-TMS2 sample. Nevertheless, similar pattern of NMR signal doubling was observed with some differences, e.g., the cross-peak doubling of E^339^ amide group from the C-terminal part of the fragment (Figure 7C, in bottom), indicating that the heterodimerization interface is analogous but somewhat shifted to the C-terminal part of the TM segment. Taken together, these results definitely show that IntPep directly interact with the NEU1 TM2 domain. Also, based on the NMR data, dimer models with almost parallel helix packing were taken for estimation of the free energy of helix association in lipid bilayer (see below).

### Calculations of the Free Energy of TM Helix-Helix Dimerization

Free energy estimation showed that IntPep-RKR forms more stable heterodimer with TM2′ (−68 ± 5 kJ/mol) than the homodimer TM2′/TM2′ itself (−54 ± 4 kJ/mol), while the heterodimer TM2′/mutIntPep-RKR is also energetically favorable, but much weaker (−39 ± 6 kJ/mol). Also, as shown in Figure 6C, heterodimers demonstrate slightly more tight packing than the wild type homodimer that can be caused with the presence of the charged RKR sequence on the C-terminus that is also involved in intermonomer contacts. Dimeric structures corresponding to the free energy minima demonstrate high similarity as shown in Figures 6D–F.

## Discussion

Besides its pivotal role in the degradation of sialyloconjugates in lysosomes known from several decades (Bonten et al., 2014), participation of NEU1 sialidase in cellular regulatory mechanisms has been discovered only recently. These major advances were concomitant with the discovery that NEU1 is also expressed at the plasma membrane of cells. By its ability to cleave sialic acid residues of membrane glycoproteins, this sialidase is involved in a wide range of human disorders as diverse as cancers, infectious and cardiovascular diseases. For instance, desialylation of the β_4_ integrin, PDGF, and IGF-1 receptors by NEU1 reduces metastasis of human colon cancer cells and decreases mitogenic signals induced by ligands (Hinek et al., 2008; Uemura et al., 2009). NEU1 also plays role in innate immunity by desialylation of TLR4 receptors enabling the removal of steric hindrance mandatory for MyD88/TLR4 association and subsequent TLR4 activation in dendritic cells and macrophages (Amith et al., 2010). More recently, desialylation of platelets by NEU1 has been shown to play critical role in platelet clearance and thrombocytopenia (Li et al., 2015). At the plasma membrane, NEU1 is also involved in the modulation of elastic fiber assembly (Hinek et al., 2006) and the biological effects mediated by the elastin-derived peptides (Wahart et al., 2019). NEU1 and its chaperone (PPCA) were both identified as components of the elastin receptor complex together with the elastin-binding protein (Hinek et al., 2006). Importantly, elastin receptor complex signaling relies on NEU1 activity (Duca et al., 2007) and inhibiting NEU1 by siRNA or by the broad-spectrum sialidase inhibitor DANA blocks signaling pathways and biological effects mediated by this receptor in different age-related vascular diseases (Blaise et al., 2013; Gayral et al., 2014; Kawecki et al., 2019). From these data, inhibition of NEU1 sialidase activity appears as a relevant pharmacological target of high-added value with potential application in cancers, infectious and cardiovascular diseases. However, no selective inhibitor of NEU1 catalytic activity is currently available and new tools are needed.

The aim of this study was to develop new tools to selectively block plasma membrane NEU1 (mNEU1) sialidase activity by interfering with its dimerization. Results from our group demonstrated that human mNEU1 behaves as a transmembrane protein at the plasma membrane of cells and identified two regions, 139–159 (TM1) and 316–333 (TM2), as potential transmembrane domains (Maurice et al., 2016). Importantly, the TM2 domain was shown to be critical for dimerization of mNEU1 and introduction of point mutations in this protein-protein interface blocked mNEU1 dimerization but also its sialidase activity (Maurice et al., 2016) pointing out the importance of this region for mNEU1 functioning. From these results, we hypothesized that interfering peptides (IntPep) able to selectively bind to the TM2 region of NEU1 should form inactive (or “weak”) IntPep-NEU1 heterodimers leading to disruption of homodimerization and decrease of sialidase activity. Indeed, as TM domains of several membrane proteins are directly involved in receptor dimerization/activation, several strategies using hydrophobic peptides which mimic the TM segments of these receptors have been developed during the last years as tools to target specifically the corresponding receptor dimer and modulate receptor activation (Albrecht et al., 2020; Westerfield and Barrera, 2020). By combining molecular dynamics (MD) simulations and experimental approaches, here we designed a number of IntPep targeting the human NEU1 TM2 region and evaluated their effects on dimerization and sialidase activity of the mNEU1. To promote delivery of the IntPep into cell membrane, two complementary strategies were used. The first one consisted of coupling the HIV-1 TAT sequence to the IntPep. The TAT peptide is the first known cell-penetrating peptide that has been widely used both for *in vitro* and *in vivo* applications (Rizzuti et al., 2015). Due to its sequence rich with positively charged lysine and arginine, the TAT peptide has been shown to efficiently deliver different cargoes into cells (Bechara and Sagan, 2013). The second strategy consisted of solubilizing the IntPep in LDS micellar solution. The efficiency of detergent micelles to carry and deliver IntPep into cells has also been widely used in a number of studies to inhibit membrane protein dimerization (Arpel et al., 2014, 2016). As already described, transmembrane peptides possess biophysical characteristics (hydrophobic) which allow rapid integration to the targeted cell membrane. Moreover, previous studies using the same delivery strategy showed that peptides using LDS micelles display both a very low immunogenicity profile and a good stability (up to 48 h at the membrane, low sensitivity to proteases when peptides are inside of the membrane) allowing their use *in vivo* and a long-term efficacy (Nasarre et al., 2010). For this second strategy, we selected the presence of the RKR motif at the C-terminus of the IntPep to optimize its orientation within the plasma membrane, as described previously (Roth et al., 2008).

The first part of the study was dedicated to the structural characterization of these IntPep. The behavior of both IntPep (TAT-IntPep, IntPep-RKR) in membrane-mimicking environment was first evaluated by MD simulations and data showed that both IntPep preserve stable helical structure and TM orientation in the POPC bilayer. Also, in membrane, they associate with the TM2 region of NEU1, and are prone to form stable IntPep/NEU1 heterodimers, indicating that the TAT sequence and RKR motif have no major incidence on the secondary structure of the IntPep and their ability to interact with its target in the membrane. Two different modeling strategies revealed a set of dimer conformations having the same residues on the dimerization interface, but varying crossing angle. Indeed, we showed with the aid of the heteronuclear NMR spectroscopy in the membrane mimetic that the membrane-embedded TM2 span E^312^–F^332^ of hNEU1, can self-associate as well as heterodimerize with the mutIntPep sequence via almost identical elongated dimerization interfaces, consistent with the homo- and heterodimerization modes having small crossing angles of the TM helices obtained by MD simulations.

Next, we evaluated the ability of FITC-TAT-IntPep and FITC-IntPep-RKR delivered from LDS micelles to reach the plasma membrane and to colocalize with mNEU1 in cells overexpressing NEU1-Flag. From our observations, colocalization areas were clearly visible at the plasma membrane between mNEU1 and the FITC-IntPep using either the TAT or the LDS approach. Furthermore, a nuclear staining is observed with FITC-TAT-IntPep likely due to the TAT sequence that harbors a nuclear localization signal (Arif et al., 2014; Smith et al., 2018). Interaction between IntPep and mNEU1 was finally validated from our co-immunoprecipitation experiments showing that FITC-IntPep co-immunoprecipitate with mNEU1 from crude membrane preparations. Taken together, these data validated our interference strategy, and the effects of both IntPep were next evaluated in experiments assessing dimerization and sialidase activity of the mNEU1. Using the both aforementioned delivery strategies, the IntPep with four point mutations previously described as inhibitors of dimerization were also tested (Maurice et al., 2016). For both formulations, a comparable and strong decrease of mNEU1 dimerization (∼50%) and sialidase activity (∼40%) was observed compared to their respective control. Moreover, for both formulations, a strong decrease of sialidase activity was observed in THP-1 derived macrophages. Even if a complete inhibition of mNEU1 sialidase activity was not observed, these results are rather promising. As previously reported, mNEU1 may contain a second transmembrane domain (TM1) (Maurice et al., 2016). Even if a lower potency for dimerization was observed for TM1 (Maurice et al., 2016), homodimerization of NEU1 through its TM1 domain is conceivable and may account for the absence of complete inhibition of mNEU1 sialidase activity. Whether NEU1 may homodimerize through TM1/TM2 domains is not known. However, these TM1/TM2 homodimers could explain the absence of a complete inhibition of mNEU1 sialidase activity by our interference strategy.

Other interesting findings came from our MD simulations of dimers formed between the NEU1 TM2 domain and IntPep. Interestingly, in the dimers, IntPep-RKR slightly change their orientation with respect to the membrane (tilt angle), arguing in favor of their reorganization to better interact with the NEU1 TM2 domain whose orientation doesn’t change upon dimerization. Evaluation of the crossing angles for TM helices argues in favor of a stable orientation of helices when IntPep-RKR and NEU1 TM2 domain are in interaction and additional stabilization comes from the introduced RKR fragment. This is confirmed by the free energy estimation showing that such a heterodimer is more stable than the native homodimer. For the TAT strategy, similar results were obtained. However, orientation changes of TAT-IntPep are less easy to quantify. These observations can be explained by the absence of structural knowledge about adding of the TAT peptide to a transmembrane helix. In contrast, no preferential value of the crossing angle was determined with the mutIntPep-RKR peptide harboring the four mutations A_319_V, G_321_I, G_328_I, and V_330_A. This heterodimer demonstrates lower structural similarity with the native dimer, and has weaker dimerization strength (higher free energy value), which is consistent with the decrease of mNEU1 dimerization inhibition observed with the mutated peptides compared to native ones. Notably, TM2/mutIntPep-RKR heterodimer is still energetically favorable, so one can expect low concentration of such a dimer to be formed.

In conclusion, we have developed an original concept to block efficiently the sialidase activity of mNEU1. By their ability to reach the plasma membrane and to bind at the dimerization interface of the TM2 domain of human NEU1 (region 316–333), these IntPep selectively and strongly decrease sialidase activity of mNEU1. Importantly, these IntPep are also strong inhibitors of membrane sialidase activity triggered by elastin-derived peptides in macrophages, known to strictly depend on NEU1 (Kawecki et al., 2019). Thus, inhibition of mNEU1 sialidase activity through specific transmembrane Intpep constitutes a promising way to decrease the deleterious effects of the elastin-derived peptides in age-related vascular diseases (Maurice et al., 2013; Duca et al., 2016) given that only a few NEU1 specific inhibitors have been reported. Thereafter, as TM peptides appear to be effective in specific targeting of dimerization and activation of mNEU1 *in vitro*, this technique could be used to inhibit *in vivo* this protein involved in several pathophysiological contexts as atherosclerosis (Gayral et al., 2014; Kawecki et al., 2019), thrombosis (Kawecki et al., 2014), insulin resistance (Blaise et al., 2013), non-alcoholic steatohepatitis (Romier et al., 2018), and cancer (Ntayi et al., 2004; Hornebeck et al., 2005; Pocza et al., 2008; Hou et al., 2016; Ren et al., 2016). In the case of cancer, as TM peptides are known to not have the ability to selectively hit the cancer cells expressing the target *in vivo*, these compounds can be combined with targeting moieties linked to nanocarriers to address this point and develop drugs with a more selective effect on a given cell type (Gamper et al., 2019).

## Data Availability Statement

The original contributions presented in the study are included in the article/Supplementary Material, further inquiries can be directed to the corresponding author/s.

## Author Contributions

All authors listed have made a substantial, direct and intellectual contribution to the work, and approved it for publication.

## Conflict of Interest

The authors declare that the research was conducted in the absence of any commercial or financial relationships that could be construed as a potential conflict of interest.

## Footnotes

1 http://model.nmr.ru/preddimer

## Supplementary Material

Supplementary Figure 1 Behavior of TM2 and interfering peptide monomers in the lipid bilayer: results of MD simulations. Time evolution of the monomer secondary structure during 250 ns MD simulations in explicit POPC membrane. **(A)** TM2 peptide, **(B)** TAT-IntPep, **(C)** IntPep-RKR, **(D)** TAT-mutIntPep, and **(E)** mutIntPep-RKR. α-helical structure is in blue, turn and bend regions are in green and yellow, 3_10_-helix is in gray, and unstructured fragments are in white. Residue numbering is shown on the left.

Supplementary Figure 2 Effects of TAT peptides and micelle-delivered peptides on cell viability. **(A,B)** 10,000 COS-7 cells were plated in 96 well plates. After 24 h, cells were incubated with TAT peptides, TAT-IntPep, nothing **(A)**, micelles containing IntPep-RKR, micelles containing mutIntPep-RKR or micelles only **(B)**. The different peptides were incubated with cells for 24 h. Results are represented compared to the no peptide control condition normalized to 100% (*n* = 3–25) (^∗∗∗^*p* < 0.001, ANOVA).

## Abbreviations

DANA: *N*-acetyl-2,3-dehydro-2-deoxyneuraminic acid
DPC: dodecylphosphocholine
EBP: elastin-binding protein
ERC: elastin receptor complex
FITC: fluorescein isothiocyanate
GPIbα: glycoprotein Ibα
IGF-1: insulin-like growth factor-1
IntPep: interfering peptide
mNEU1: membrane NEU1
LDS: lithium dodecyl sulfate
mutIntPep: mutant interfering peptide
Myd88: myeloid differentiation primary response 88
NEU: neuraminidase
PDGF: platelet-derived growth factor
POPC: 1-palmitoyl-2-oleoyl-phosphatidylcholine
PPCA: protective protein/cathepsin A
PepR: recombinant peptide
TAT: transactivator of transcription
TM: transmembrane
TLR4: toll like receptor 4 transmembrane
TMS: transmembrane segment.
