# Supplementary figures and images for: Transmembrane Peptides as a New Strategy to Inhibit Neuraminidase-1 Activation
Source: Front Cell Dev Biol. 2020 Dec 16;8:611121. doi: 10.3389/fcell.2020.611121 (PMC7772355; doi:10.3389/fcell.2020.611121)

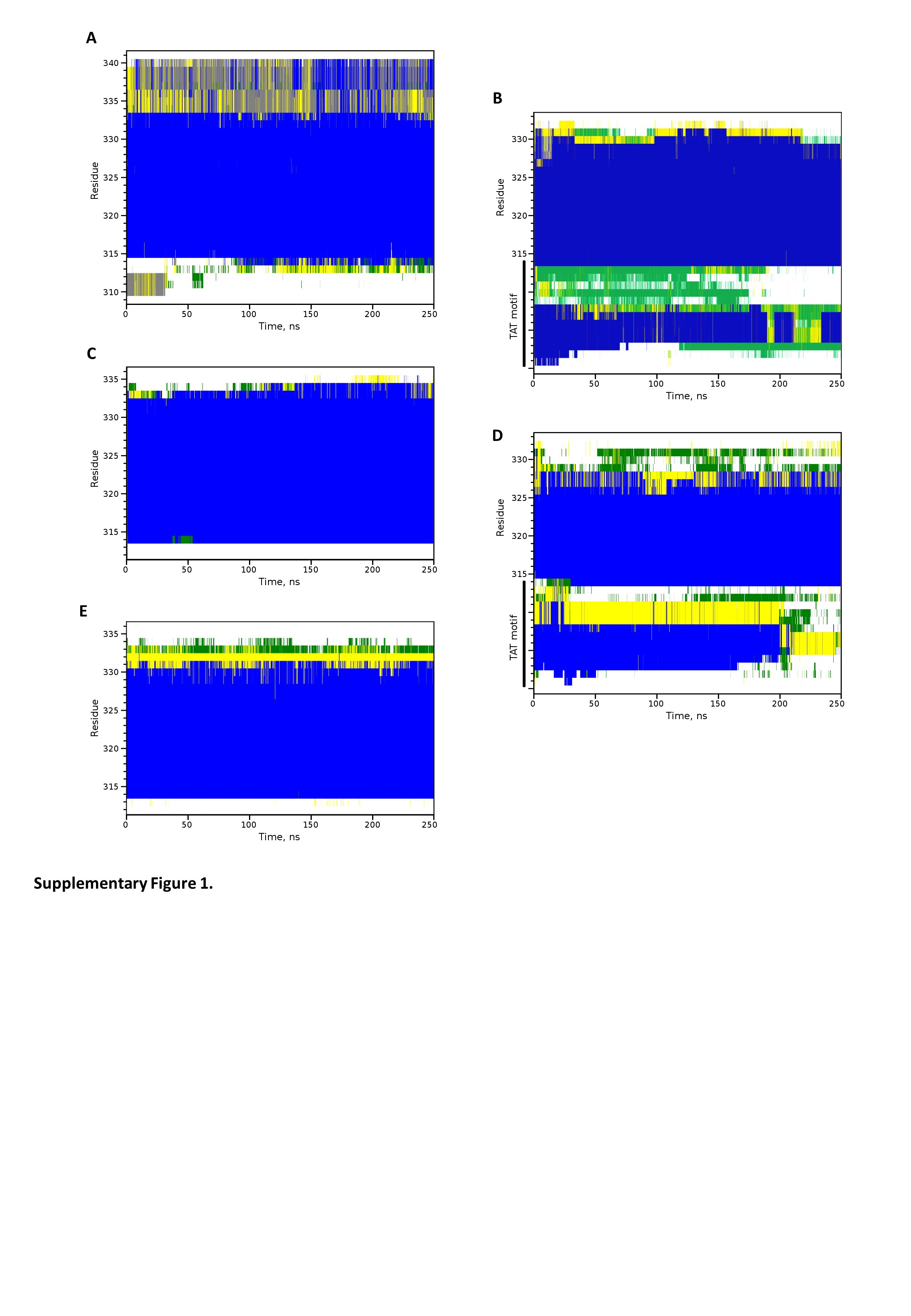

Supplement: Supplementary Figure 1 — Behavior of TM2 and interfering peptide monomers in the lipid bilayer: results of MD simulations. Time evolution of the monomer secondary structure during 250 ns MD simulations in explicit POPC membrane. (A) TM2 peptide, (B) TAT-IntPep, (C) IntPep-RKR, (D) TAT-mutIntPep, and (E) mutIntPep-RKR. α-helical structure is in blue, turn and bend regions are in green and yellow, 310-helix is in gray, and unstructured fragments are in white. Residue numbering is shown on the left. [file Image_1.jpg]

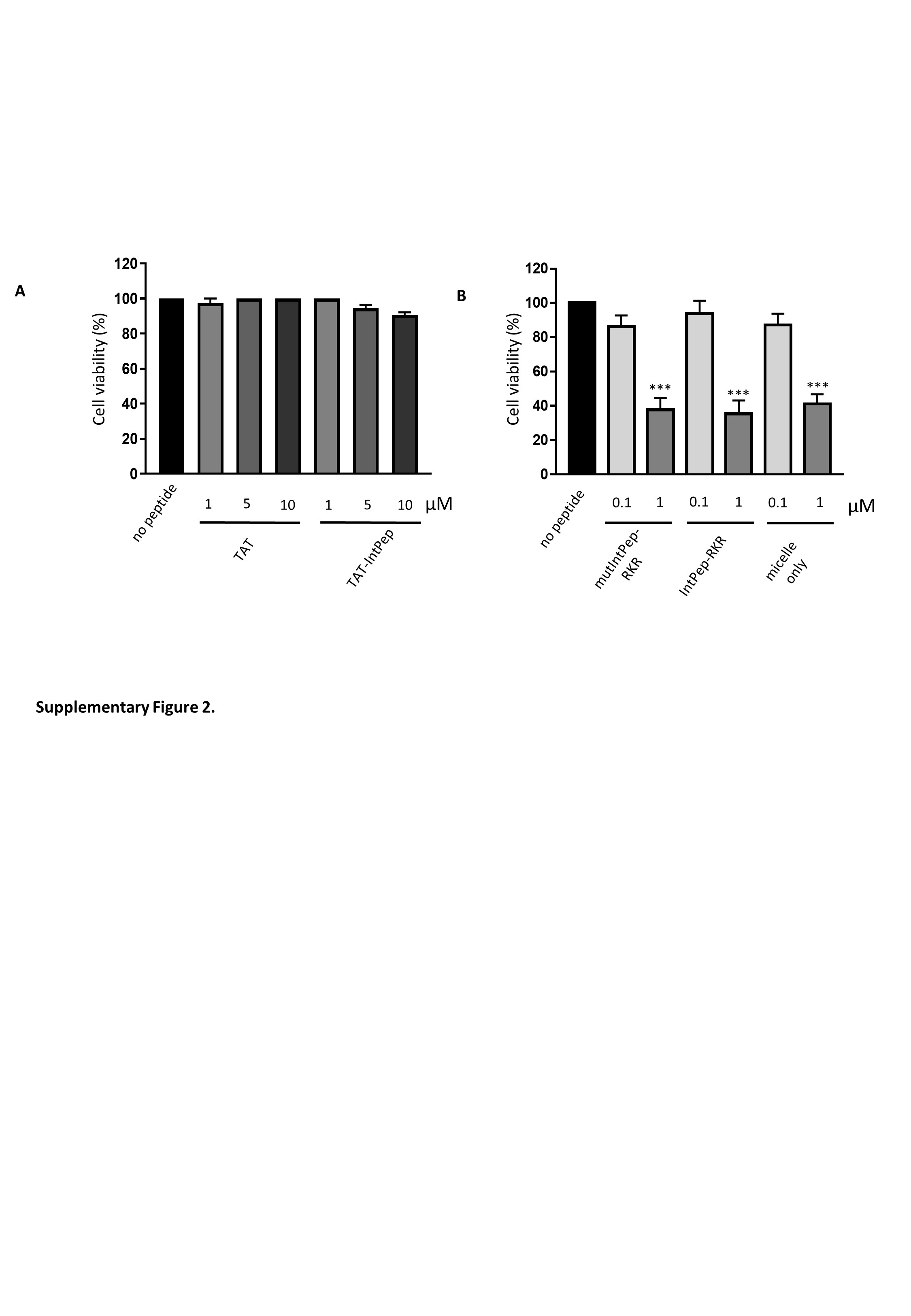

Supplement: Supplementary Figure 2 — Effects of TAT peptides and micelle-delivered peptides on cell viability. (A,B) 10,000 COS-7 cells were plated in 96 well plates. After 24 h, cells were incubated with TAT peptides, TAT-IntPep, nothing (A), micelles containing IntPep-RKR, micelles containing mutIntPep-RKR or micelles only (B). The different peptides were incubated with cells for 24 h. Results are represented compared to the no peptide control condition normalized to 100% (n = 3–25) (∗∗∗p < 0.001, ANOVA). [file Image_2.jpg]
